# Black Raspberries and Protocatechuic Acid Mitigate DNFB-Induced Contact Hypersensitivity by Down-Regulating Dendritic Cell Activation and Inhibiting Mediators of Effector Responses

**DOI:** 10.3390/nu12061701

**Published:** 2020-06-06

**Authors:** Kelvin Anderson, Nathan Ryan, Arham Siddiqui, Travis Pero, Greta Volpedo, Jessica L. Cooperstone, Steve Oghumu

**Affiliations:** 1 Department of Pathology, The Ohio State University Wexner Medical Center, Columbus, OH 43210, USA; anderson.2325@osu.edu (K.A.); ryan.1046@osu.edu (N.R.); siddiqui.120@osu.edu (A.S.); pero.17@osu.edu (T.P.); volpedo.1@osu.edu (G.V.); 2 Division of Anatomy, The Ohio State University Wexner Medical Center, Columbus, OH 43210, USA; 3 College of Dentistry, The Ohio State University, Columbus, OH 43210, USA; 4 Department of Microbiology, The Ohio State University, Columbus, OH 43210, USA; 5 Department of Horticulture and Crop Science, The Ohio State University, Columbus, OH 43210, USA; cooperstone.1@osu.edu; 6 Department of Food Science and Technology, The Ohio State University, Columbus, OH 43210, USA

**Keywords:** *Rubus occidentalis*, contact hypersensitivity, inflammatory, IFN-γ, dendritic cells

## Abstract

Contact hypersensitivity (CHS) is the most common occupational dermatological disease. Dendritic cells (DCs) mediate the sensitization stage of CHS, while T-cells facilitate the effector mechanisms that drive CHS. Black raspberry (*Rubus occidentalis*, BRB) and BRB phytochemicals possess immunomodulatory properties, but their dietary effects on CHS are unknown. We examined the effects of diets containing BRB and protocatechuic acid (PCA, a constituent of BRB and an anthocyanin metabolite produced largely by gut microbes), on CHS, using a model induced by 2,4-dinitrofluorobenze (DNFB). Mice were fed control diet or diets supplemented with BRB or PCA. In vitro bone-marrow derived DCs and RAW264.7 macrophages were treated with BRB extract and PCA. Mice fed BRB or PCA supplemented diets displayed decreased DNFB-induced ear swelling, marked by decreased splenic DC accumulation. BRB extract diminished DC maturation associated with reduced *Cd80* expression and Interleukin (IL)-12 secretion, and PCA reduced IL-12. Dietary supplementation with BRB and PCA induced differential decreases in IL-12-driven CHS mediators, including Interferon (IFN)-γ and IL-17 production by T-cells. BRB extracts and PCA directly attenuated CHS-promoting macrophage activity mediated by nitric oxide and IL-12. Our results demonstrate that BRB and PCA mitigate CHS pathology, providing a rationale for CHS alleviation via dietary supplementation with BRB or BRB derived anthocyanins.

## 1. Introduction

Contact hypersensitivity (CHS) is a delayed hypersensitivity response to physical contact with normally tolerated antigens. Clinically, CHS presents acutely as a dermatitis marked by redness, scaling, pruritus and/or swelling. Chronic CHS is indicated by lichenification, fissuring or scaling [1]. These symptoms cause itchiness, burning pain and general discomfort in patients, which can disrupt mood, behavior and sleep, resulting in impaired quality of life [2,3]. CHS is also the most common occupational dermatological disease, with a reported incidence of 15–20% in the general adult population, and is known to be triggered by many workplace and lifestyle items, including rubber gloves, jewelry, personal care items, fragrances, dyes and detergents [1,4]. Currently, when triggers cannot be avoided, CHS is primarily treated and managed through topical or oral corticosteroids, however, these medications do not guarantee clinical remission and may lead to well-documented adverse effects, including immune deficiency, site-specific skin atrophy, weight gain and osteoporosis [5,6].

CHS is induced by haptens, antigens that become immunogenic when bound to carrier proteins in vulnerable individuals, which stimulate antigen presenting cells (APCs) to induce an adaptive immune response. Repeated exposure to haptens triggers the migration of activated T-cells to the site of exposure, where they induce an inflammatory response, driving CHS pathogenesis [7]. CHS occurs in two phases: (1) the sensitization phase is the initial stage in which individuals become sensitized to an antigen and is mediated by APCs; (2) the elicitation phase which follows sensitization and is induced upon subsequent exposure to the antigen. The elicitation phase is a T-cell mediated response and is responsible for the pathologic effects seen in CHS [7]. Specifically, antigen-specific Th1 cells elicit an inflammatory Type 1 immune response, driving the pathogenesis of CHS through the activation of effectors such as macrophages via cytokines, primarily Interferon (IFN)-γ [8,9,10]. IFN-γ production by CD8^+^ cells has also been implicated as a major factor in CHS [11]. 

Recent studies have shown that diet plays a significant role in mitigating the development of allergic illnesses, with the consumption of antioxidant rich foods shown to be particularly efficacious in reducing allergic responses [12,13]. Interestingly, murine models of CHS using 2.4-dinitrofluorobenze (DNFB), which recapitulates the human disease, show that the consumption of soy-derived, and carotenoid rich foods, may improve CHS pathology [14,15,16,17]. Black raspberries (BRB; *Rubus occidentalis*) contain a wide array of bioactive phytochemicals, such as anthocyanins, quercetin, ellagic acid, and β-sistosterol, that possess anti-inflammatory and immunomodulatory properties [18,19,20,21]. These components are thought to be the basis for BRB’s observed ability to inhibit chronic inflammation [22] and oxidative stress [23]. These studies suggest that BRB and their bioactive components may possess therapeutic effects in diseases associated with inflammatory immune dysregulation. Anthocyanins in particular draw interest due to their high concentration in BRB and evidence of their antioxidant activity [24]. The three most abundant anthocyanins in BRB are cyanidin-3-*O*-glucoside, cyanidin-3-*O*-rutinoside, and cyanidin-3-*O*-(2^G^-xylosylrutinoside) [25]. Protocatechuic acid (PCA), the most abundant of several small and bioavailable gut microbial metabolites of anthocyanins [26], accounts for over 70% of the metabolites of BRB anthocyanins in humans, and has been identified as a potential driver of BRB mediated immunomodulatory effects [27]. PCA has been demonstrated to be bioavailable in the plasma and various tissues, such as the colon and liver, after consumption of BRB, and has been shown to elicit therapeutic properties in inflammation associated diseases [28,29,30]. Parenteral administration of PCA has also been found to reduce acute airway inflammation and sequalae in vivo, in a murine model of allergic asthma [31]. However, the potential effect of dietary administration of BRB and PCA on CHS remain uncharacterized. 

In this study, we investigated the impact of dietary administration of BRB or PCA on CHS, using an in vivo murine model of CHS induced by DNFB. We examined the effects of these diets on CHS dermal swelling and characterized the molecular and immunological mechanisms associated with BRB and PCA mediated inhibition of CHS. Our results indicate that diets supplemented with BRB or PCA are effective in mitigating the pathology of CHS.

## 2. Materials and Methods 

### 2.1. Animal Handling

Female BALB/c mice aged 6 weeks old were purchased from Jackson Laboratories (Bar Harbor, MI, USA). All mice were housed in groups of 5 and maintained with a 12 h day/night cycle and access to food and water ad libitum, under a standard temperature range of 20–24 °C and relative humidity between 40 and 60%. Animals were kept in accordance with regulations maintained by University Laboratory Animal Resources and were approved by the Institutional Animal Care and Use Committee (Protocol #2018A00000054), and the Institutional Biosafety Committee of The Ohio State University.

### 2.2. Animal Diets

Mice were randomized into four groups based on diet and DNFB sensitization, beginning three weeks prior to sensitization; standardized minimal nutrient rodent chow AIN-76A sensitized with vehicle only (*n* = 5), minimal nutrient rodent chow AIN-76A sensitized with DNFB (*n* = 5), AIN-76A supplemented with 5% w/w freeze-dried black raspberry (BRB) powder sensitized with DNFB (*n* = 5), or AIN-76A supplemented with 500 ppm protocatechuic acid (PCA, Acros Organics, NJ, USA) sensitized with DNFB (*n* = 5). Whole BRB powder was used in this study to reflect what is typically consumed in a diet. Anthocyanin content of the BRB powder used for diet preparation was 27.7 mg cyanidin-3 glucoside equivalents/g. The PCA dose was selected to match, on a molar basis, the amount of total anthocyanins found in the 5% BRB diet. Based on previous studies [32], we estimate the amount of PCA in our BRB diet [formulated at 5% weight/weight (w/w)] to be about 4 mg/kg diet. The 5% BRB diet contained 3.08 mmol total anthocyanins, or 1.39 g total anthocyanins per kg diet, as determined using the pH differential method [33]. Animals were maintained on their individual diets throughout the duration of the experiment. BRB used in this feed was purchased from the Stokes Berry Farm (Wilmington, OH, USA), before being shipped to Van Drunen Farms (Momence, IL, USA) for freeze drying. Standard AIN-76A and its special formulations were prepared by Dyets Inc. (Bethlehem, PA, USA), and stored at −20 °C, before being provided to the animals ad libitum (Table 1) [34]. A separate extended feeding study was performed to determine mouse weights and food intake, which showed no differences between the prepared diets (Figure S1A,B).

### 2.3. Black Raspberry Extract Preparation for In Vitro Studies

Black raspberry powder was extracted three times with 80:19:1 ethanol/water/formic acid, sonicated for 10 min in an ice bath, and vacuum filtered. The resulting extract was concentrated on a rotary evaporator and subsequently lyophilized to remove remaining water. This procedure yielded 40 g extract/100 g black raspberry powder and an anthocyanin content of 57 mg cyanidin-3-glucoside equivalents/g extract. An analysis of anthocyanins in the BRB was performed according to protocols described previously [33].

### 2.4. Induction of Contact Hypersensitivity

After 3 weeks under their respective diets, contact hypersensitivity was induced by the abdominal application of 0.5% DNFB (Acros Organics, NJ, USA) and dissolved in a 4:1 olive oil-acetone vehicle or vehicle alone, as described previously [35]. Mice were sensitized in this way on days 0, 4 and 7. On days 14 and 17, mice were challenged with an application of 20 µL 0.2% DNFB solution or vehicle alone on the dorsal surface of the left ear. Ear swelling was determined by measurement with electronic calipers 24 h after challenge 1 and 24 h after challenge 2, with percentage swelling determined by dividing the difference in thickness of the challenged left ear and unchallenged right ear by the thickness of the unchallenged right ear. All measurements were taken 10 times from each mouse, with the average of these measurements used to determine ear width. Animals were euthanized on day 18, which was 24 h after challenge 2 (Figure S1C).

### 2.5. Flow Cytometry

Upon terminal sacrifice, spleens and draining lymph nodes of all mice were passed through a 70 µm nylon mesh to generate single cell suspension. Cells were incubated with fluorochrome conjugated antibodies targeting CD3, CD4, CD8, CD11b, CD11c, CD49b, CD80, CD86, Ly6C, Ly6G, F4/80, and MHCII. Additionally, cells were intracellularly stained for IFN-γ after PMA and ionomycin cellular activation (BioLegend, San Jose, CA, USA). All flow cytometric data analyses were performed using the FlowJo software (Tree Star Inc., Ashland, OR, USA).

### 2.6. Histopathology

Ear tissue was collected at terminal sacrifice and fixed for 24 h in 10% neutral buffered formalin. After fixation, the ears were embedded in paraffin blocks, from which 5-µm sections were cut. Sections were stained with hematoxylin and eosin and examined microscopically. The widths of the ear sections were measured in a blind fashion under the microscope using the Leica Application Suite (Leica Biosystems Inc., Buffalo Grove, IL, USA). An increase in ear thickness was determined as the difference between the thickness of the challenged left ear and the thickness of the unchallenged right ear of each mouse.

### 2.7. LPS Stimulation of RAW264.7 Macrophages, and Bone Marrow-Derived Macrophages and Dendritic Cells

Eight-week-old BALB/c mice were used for dendritic cell isolation. Tibias and femurs were harvested from euthanized mice for the collection of bone marrow cells. Cells were treated with ACK lysis buffer and placed in 24 well plates at a concentration of 5.0 × 10^4^ cells/well in complete RPMI medium, supplemented with 10% fetal bovine serum (Atlanta Biologicals), 1% penicillin (20 Units/mL)/streptomycin (20 μg/mL) (Life Technologies) and RPMI supplemented with 25 ng/mL recombinant mouse granulocyte macrophage colony stimulating factor (GM-CSF) [36]. RAW264.7 macrophages were grown to 70% confluence and seeded at a density of 5 × 10^5^ cells/well in a 12-well plate at a volume 500 µL. All cells were incubated at 37 °C and 5.0% CO_2_. Next, the cells were randomly divided into groups receiving no treatment, or receiving one of the following treatments: black raspberry extract (BRB-E, at concentrations of 50 µg/mL or 5 µg/mL, dissolved in PBS), or protocatechuic acid (Acros Organics, NJ, USA) (PCA, at concentrations of 50 µM, and 5 µM, dissolved in DMSO), in a total volume of 1 mL per well. These treatments were added before differentiation of bone marrow-derived cells to wells designated for ex vivo differentiation studies, after the differentiation of bone-marrow derived cells in wells designated for activation studies, and 8 h after seeding RAW264.7 macrophages. All wells contained a final volume of 0.125% DMSO. In the case of activation study bone-marrow derived cells and RAW264.7 macrophages, half of the samples from each group were stimulated with 1 µg/mL lipopolysaccharide (LPS), following overnight incubation with the treatments for 12, 24, and 96 h. Differentiation study bone-marrow derived cells were given LPS simultaneously with the activation study cells. Following LPS stimulation, cell supernatants were obtained for cytokine ELISA and cell lysates were harvested for RTq PCR gene expression analysis.

### 2.8. Real Time Quantitative PCR

Cellular RNA was obtained directly from lysates using guanidinium thiocyanate-phenol-chloroform extraction. All RNA was stored at −80 °C prior to cDNA synthesis using the High Capacity cDNA Reverse Transcription Kit (Applied Biosystems, Foster City, CA, USA). Synthesized cDNA were then amplified by PCR using the PowerUp SYBR Green Master Mix (BioRad, Hercules, CA, USA) and primers designed using the IDT RealTime qPCR Tool (https://www.idtdna.com/scitools/Applications/RealTimePCR/, Integrated DNA Technologies, Coralville, IA, USA). Then, β-actin (*Actb*) was used as our reference gene against *Cd80*, *Cd86*, *Il12b*, *Inos*, and *Mhcii*. Fold induction was calculated using the 2^−ΔΔCt^ method.

### 2.9. Enzyme-Linked Immunosorbent Assay

Single cell suspensions were prepared from spleens and lymph nodes. Cells were incubated with plate bound αCD3 (1 μg/mL) and αCD28 (1 μg/mL) antibodies (Biolegend, SanDiego, CA, USA). Cells of the draining lymph nodes were incubated for 24 h, while spleen-derived cells were incubated for 72 h. Supernatants collected from single cell suspensions generated using mouse lymph nodes or spleens were assayed for cytokines IFNγ, IL-12 and IL-17. Antibodies used for ELISA were purchased from BioLegend (San Diego, CA, USA). Supernatants collected from macrophage and bone-marrow derived cells were assayed for IL-12. Capture and detection antibodies were purchased from Biolegend (San Diego, CA, USA).

### 2.10. Griess and Cytotoxicity Assay

The potential cytotoxicity of varying BRB-E and PCA treatment concentrations and DMSO at a standardized DMSO concentration of 0.125% was assessed by measuring lactose dehydrogenase (LDH) in cell supernatants, using the Pierce LDH cytotoxicity assay kit (ThermoScientific, Rockford, IL, USA). Percent cytotoxicity was determined based on an equivalent sample wholly lysed using the kit’s included lysis buffer. NO production by RAW264.7 macrophages and bone marrow-derived dendritic cells was determined by performing a Griess assay using Griess reagent (0.2% naphthylethylenediamine dihydrochloride, and 2% sulphanilamide in 5% phosphoric acid), with NO concentrations approximated based on a 0.1 M sodium nitrite standard.

### 2.11. Statistical Analysis

All statistical analyses were performed using GraphPad Prism software v8.0.2 (GraphPad Software, San Diego, CA, USA). Unpaired, two-tailed Student’s *t* tests were performed between groups to determine statistical significance of difference, with a *p*-value threshold of 0.05.

## 3. Results and Discussion

### 3.1. Auricular Inflammation Is Attenuated in Mice Fed with BRB and PCA Supplemented Diets during DNFB-Induced CHS

**Figure 1:**
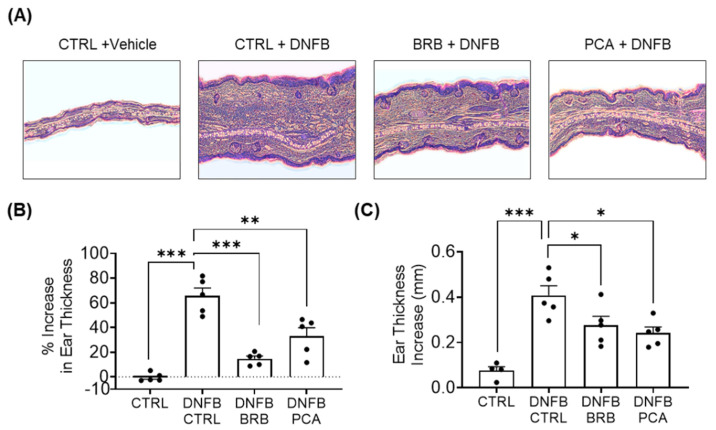
Auricular Inflammation is Attenuated in Mice Fed BRB and PCA Supplemented Diets During 2,4-dinitrofluorobenze (DNFB)-Induced contact hypersensitivity (CHS), with BRB displaying the strongest effects. (**A**) H&E stained cross sections of the challenged left ears taken from representative experimental mice at terminal sacrifice. (**B**) The percent increase in swelling of the left ears experimental mice 24 h following challenges 1 and 2. Measurements were determined by dividing the difference in ear size between the left challenged ear measurement and unchallenged right ear measurement, by the unchallenged right ear measurement. (**C**) Increase in ear thickness determined by taking the difference between individual left ear thickness and the unchallenged average right ear thickness of the respective group. * *p*-value ≤ 0.05; ** *p*-value < 0.01; *** *p*-value < 0.005 for comparisons between DNFB-challenged mice fed control diet and comparison groups using Student’s *t* test. Black circles represent individual mice. CTRL—control diet; BRB—black raspberry diet; PCA—protocatechuic acid diet.

We induced CHS using DNFB in a well characterized murine model (Figure S1). As expected, DNFB-challenged mice experienced histopathological changes analogous to human CHS (Figure 1A). Compared to DNFB-challenged mice fed control diet, DNFB-challenged mice fed diets supplemented with BRB and PCA experienced reduced swelling in the challenged ear (Figure 1A,B). A histopathological analysis performed on the DNFB-challenged ears supported these observations, showing that DNFB-challenged mice fed diets supplemented with BRB or PCA showed decreased swelling compared to challenged mice fed control diet (Figure 1C). Taken together, our results demonstrate that dietary administration of BRB and PCA decreases swelling in an experimental model of DNFB-induced CHS. These data also suggest that anthocyanin derived metabolites and other BRB phytochemicals are major drivers of the BRB mediated reduction of DNFB-induced CHS.

### 3.2. Effects of BRB and PCA on Dendritic Cell Migration, Maturation and Antigen Presentation during DNFB Induced CHS

**Figure 2:**
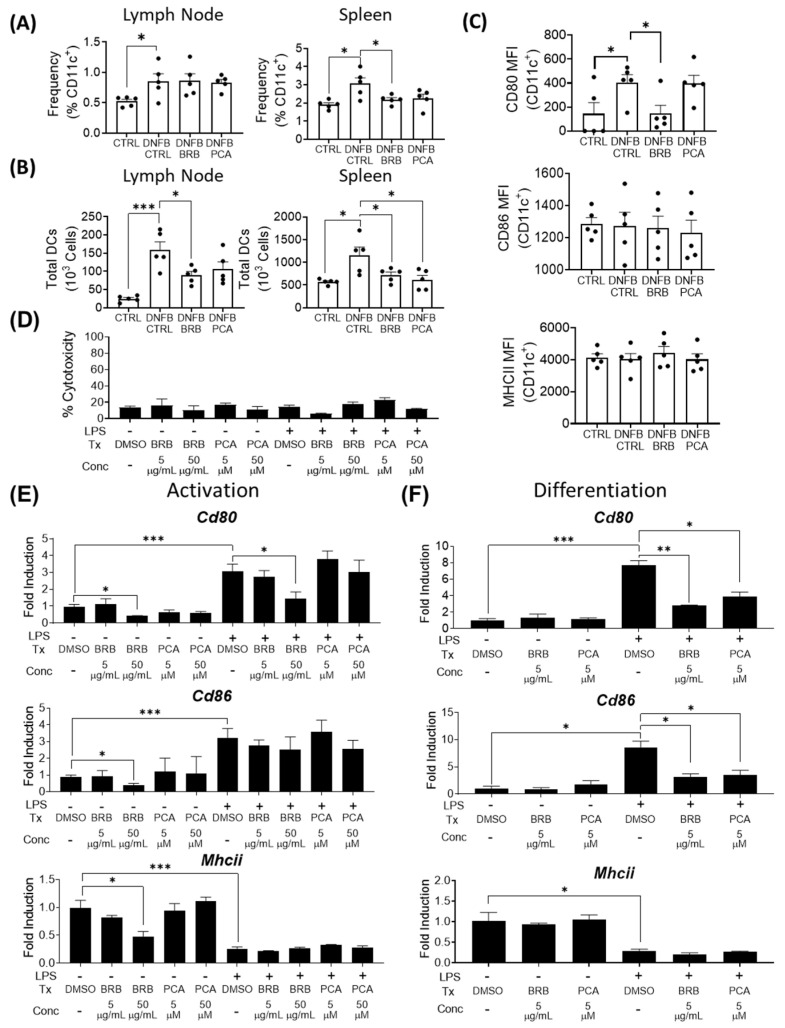
Effects of BRB and PCA on dendritic cell migration, maturation and antigen presentation during DNFB induced CHS (**A**) CD11c^+^ dendritic cell population frequencies among total live cells within the draining lymph nodes and spleens of experimental mice determined by flow cytometry. (**B**) Total dendritic cell counts within the spleens and lymph nodes measured as the product of CD11c^+^ dendritic cell population frequencies and hemocytometer-derived cell counts of whole organ single cell suspensions. (**C**) Mean fluorescent intensity (MFI) of CD80, CD86, and MHCII expression by splenic CD11c^+^ dendritic cells. (**D**) Percent cytotoxicity, relative to a wholly lysed equivalent sample, by experimental concentrations of DMSO, BRB-E, and PCA, measured by an LDH cytotoxicity assay. (**E**,**F**) Expression of *Cd80*, *Cd86*, and *Mhcii* by (**E**) activation study and (**F**) differentiation study dendritic cells determined by RTqPCR. * *p*-value < 0.05; ** *p*-value < 0.01; *** *p*-value < 0.005 for comparisons between DNFB-challenged mice fed control diet and comparison groups using Student’s *t* test, and for comparisons between DMSO control samples and comparison groups.

In the sensitization phase of DNFB-induced CHS, antigen is captured by dendritic cells, which migrate to secondary lymphoid organs, such as the regional lymph nodes and spleen, to initiate cell-mediated immunity [4]. To determine the effects of BRB and PCA on this phase of the immune response to DNFB-mediated CHS, we analyzed dendritic cell populations in the lymph nodes and spleen by flow cytometry (Figure S2). As expected, we saw an elevation of dendritic cells in the lymph nodes and spleens of DNFB-induced mice fed control diet compared to the non-DNFB-sensitized mice. In the lymph node, CD11c^+^ dendritic cell populations did not vary significantly between DNFB-challenged mice fed control diet and DNFB-challenged mice fed diets supplemented with BRB or PCA (Figure 2A). Since the spleen is a major site involved in generating immunological responses to DNFB mediated CHS, we examined dendritic cell populations in this secondary lymphoid organ [37]. Interestingly, we observed a significant reduction in splenic CD11c^+^ dendritic cell accumulation in DNFB challenged mice fed BRB supplemented diets, compared to DNFB-challenged mice fed the control diet. A PCA supplemented diet also led to a reduction, though this was not statistically significant (Figure 2A). We also determined the total number of dendritic cells in both the spleens and lymph nodes [38,39], which we suspected to be decreased by BRB diets compared to the mice fed control diet. Using cell counts determined from whole organ lysates and our calculated CD11c^+^ frequencies from flow cytometry, we found that mice with BRB supplemented diets showed a significantly decreased overall dendritic cell population in their draining lymph nodes compared to the mice fed control diet. Mice fed a PCA supplemented diet showed, as well, decreased total dendritic cell populations in the draining lymph nodes, though this did not reach statistical significance. In the spleens, both BRB and PCA fed mice demonstrated a significantly lower number of dendritic cells compared to mice fed control diet (Figure 2B). These data show that BRB and PCA dietary supplementation inhibit dendritic cell migration to splenic sites during DNFB-mediated CHS, while BRB, but not PCA, leads to decreased dendritic cell infiltration into the lymph nodes.

Next, we determined the impact of BRB and PCA on dendritic cell maturation. During CHS, dendritic cells uptake antigen, migrate to secondary lymphoid organs, and acquire antigen presenting and capabilities, marked by expression of the antigen presentation marker MHCII and co-stimulatory molecules CD80 and CD86 [40,41]. We observed that the expression of the costimulatory molecule CD80, which was elevated in DNFB-challenged mice, was significantly reduced in the spleens of DNFB-challenged mice fed a BRB supplemented diet, but not in DNFB-challenged mice fed a PCA supplemented diet (Figure 2C, Figure S2B). No changes were observed in the mean fluorescent intensity (MFI) of the dendritic cell expression of MHCII and CD86 between DNFB-challenged mice fed control, BRB or PCA supplemented diets (Figure 2C, Figure S2B). These data suggest that the reduction of dendritic cell co-stimulatory molecule expression by BRB phytochemicals is not mediated by PCA. However, the reduction of CD80 expression in dendritic cells by PCA is not required for its inhibition of inflammation during experimental CHS.

Given the effects on dendritic cells observed in DNFB-challenged mice fed diets supplemented with BRB or PCA, we performed a more in-depth analysis of dendritic cell immuno-modulation by these compounds. Specifically, we examined whether black raspberry extract (BRB-E) and PCA could affect the differentiation of hematopoietic stem cells into dendritic cells and the activation of bone-marrow derived dendritic cells in vitro. To ensure that experimental concentrations were not cytotoxic to dendritic cells, we determined cell cytotoxicity using an LDH cytotoxicity assay. Concentrations of BRB-E and PCA used for our studies showed no significant cytotoxic effects on dendritic cells (Figure 2D). In both differentiation and activation studies, we measured the expression of *Cd80*, *Cd86*, and *Mhcii* via RTqPCR. Corroborating our in vivo findings, our activation study of dendritic cells displayed a down-regulation of *CD80* expression in LPS-stimulated cells treated with BRB-E at a concentration of 50 µg/mL, compared to the LPS-stimulated DMSO control cells. Additionally, we observed a significant decrease in *Cd80*, *Cd86*, and *Mhcii* expression in non-LPS stimulated cells treated with 50 µg/mL BRB-E, compared to the non-LPS stimulated DMSO control cells. These findings were not observed in cells treated with 5µg/mL BRB-E or in PCA-treated samples, indicating a dose-dependent effect of BRB on the antigen-presentation ability by dendritic cells that is independent of PCA (Figure 2E). Our differentiation study showed that 5µg/mL BRB-E was able to reduce the expression of *Cd86* in addition to *Cd80*. Moreover, 5 µM PCA treatment also down-regulated the expression of these costimulatory markers in a dose dependent manner compared to the LPS-stimulated DMSO control cells. Furthermore 50 µg/mL BRB-E treated cells were inviable for analysis, likely due to extended exposure (Figure 2F).

Taken together, our data indicates that a component of BRB other than PCA is potentially responsible for the down-regulation of dendritic cell (DC) maturation and DNFB sensitization. However, our data suggest that PCA influences bone-marrow derived hematopoietic stem cell differentiation into dendritic cells that may have contributed to the decreased splenic dendritic cell populations observed in DNFB-challenged mice fed a PCA supplemented diet.

### 3.3. IL-12, a Key Mediator of DNFB-Induced CHS Is Reduced by BRB-E and PCA

**Figure 3:**
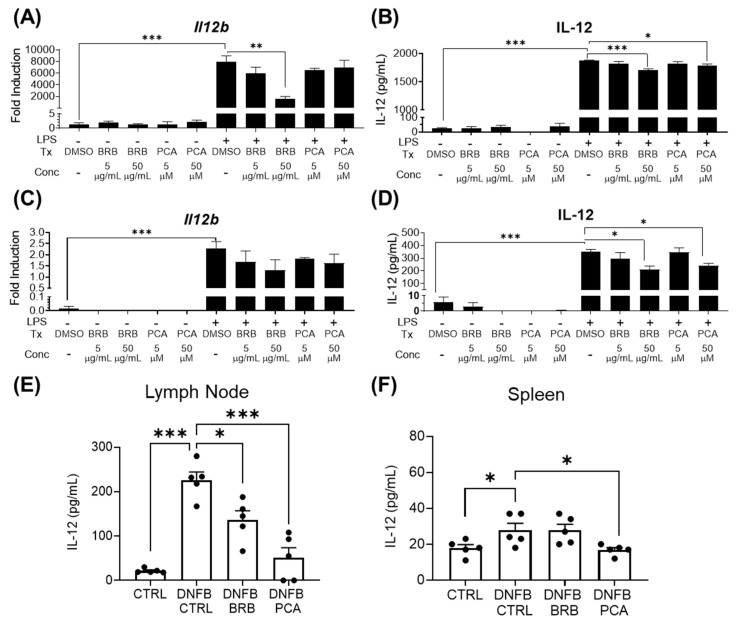
IL-12, a key mediator of DNFB-induced CHS, is reduced by black raspberry extract (BRB-E) and PCA. (**A**) Fold induction of *Il12b* expression in activated bone marrow-derived dendritic cells at 24 h after LPS stimulation. (**B**) IL-12 production by activation study bone marrow-derived dendritic cells, 24 h after LPS stimulation. (**C**) Fold induction of *Il12b* expression by RAW264.7 macrophages after 12 h after LPS stimulation. (**D**) IL-12 production by RAW264.7 macrophages after 24 h of LPS stimulation. (**E**,**F**) IL-12 production (pg/mL) by cells taken from the (**E**) draining lymph nodes after 24 h of CD3 stimulation and (**F**) spleen after 12 h of CD3 stimulation. * *p*-value < 0.05; ** *p*-value < 0.01; *** *p*-value < 0.005 for comparisons between DNFB-challenged mice fed control diet and comparison groups using Student’s *t* test, and for comparisons between DMSO control samples and comparison groups.

IL-12 is a key mediator of CHS, driving antigen presentation, costimulatory molecule expression [42] and promoting IFN-γ production in T-lymphocytes and NK cells [43,44]. The suppression of IL-12 has been shown to preclude sensitization to DNFB [45]. Therefore, we analyzed the impact of BRB-E and PCA on IL-12 production by dendritic cells. After LPS stimulation, the *Il12b* gene expression by dendritic cells was significantly diminished in cells treated with BRB-E in a dose dependent manner, compared to LPS-stimulated untreated cells. No differences in *Il12b* gene expression was observed in PCA-treated cells in our activation study (Figure 3A). The suppression of dendritic cell mediated IL-12 production by BRB-E was corroborated by ELISA analysis. Interestingly, 50 µM PCA was found to significantly down-regulate IL-12 secretion at 24 h, though to a lesser extent than BRB-E, indicating that PCA has a minimal effect on the ability of dendritic cells to secrete IL-12 after LPS stimulation (Figure 3B). These results are similar to the relative effects of BRB and PCA on *Cd80* expression by LPS activated dendritic cells and CD80 expression by the splenic dendritic cells of DNFB-challenged mice fed BRB supplemented diets.

IL-12 production by macrophages also promotes Th1 differentiation and response [46], leading us to investigate *Il12b* expression and IL-12 secretion by LPS-stimulated RAW264.7 macrophages treated with BRB-E or PCA. Compared to control cells, BRB-E and PCA reduced *Il12b* expression in macrophages (Figure 3C). IL-12 protein secretion, as determined by ELISA, was also significantly decreased in LPS stimulated macrophages by BRB-E and PCA in a dose dependent manner (Figure 3D). To corroborate the results of our in vitro activation studies, we analyzed IL-12 cytokine production in lymph nodes and spleens of our in vivo model of DNFB-induced CHS. In DNFB-challenged mice fed BRB-E and PCA supplemented diets, we observed a significant decrease in IL-12 production by lymph node cells, but not spleen cells, compared to the DNFB-challenged mice fed a control diet (Figure 3E,F). Given the stronger effect of PCA on macrophages compared to dendritic cells, our data suggest that the inhibition of IL-12 production in the lymph nodes of DNFB-challenged mice fed PCA supplemented diets is due to direct effects on macrophages, rather than dendritic cells.

The effects of PCA and BRB on IL-12 production depending on cell type reveal the independent pathways that are impacted during CHS inhibition. BRB (which contain a wide array of bioactive anti-inflammatory compounds) possess a potent ability to suppress IL-12 production by dendritic cells, while the effects of PCA on dendritic cell IL-12 production was minimal. This suggests that other anthocyanin derived compounds other than PCA, or non-anthocyanin derived compounds, in BRB likely drive this reduction. On the other hand, BRB and PCA treatments similarly down-regulate IL-12 production by macrophages, indicating that anthocyanin derived metabolites in BRB are likely major players in this effect. These observations may be in large part due to the contrasting regulation of IL-12 production by dendritic cells and macrophages [47].

### 3.4. IFN-γ Production Is Attenuated in DNFB Challenged Mice Fed BRB and, to a Lesser Extent, PCA Supplemented Diets

**Figure 4:**
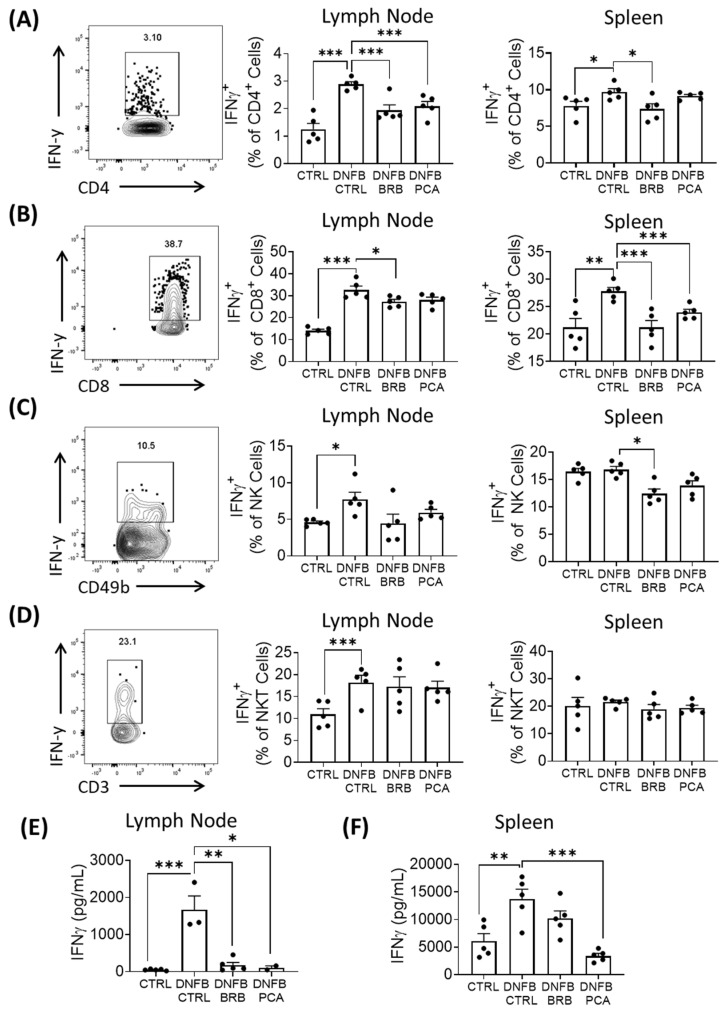
IFN-γ production is attenuated in DNFB challenged mice fed BRB and, to a lesser extent, PCA supplemented diets. (**A**,**B**) Frequency of IFN-γ expression by (**A**) CD4^+^ cells, (**B**) CD8^+^ cells, (**C**) CD49b^+^CD3^−^ NK cell, and (**D**) CD49b^+^CD3^+^ NKT cells within the draining lymph nodes and spleens of experimental mice, determined by flow cytometry. Representative graphs of the gated populations are shown. (**E**,**F**) Production of IFNγ by CD3 stimulated T-cells, isolated from the lymph nodes and spleens. Concentrations in the supernatants of plated T-cells were determined by ELISA after 24-h incubation for lymph nodal populations, and after 72 h incubations for splenic populations. * *p*-value < 0.05; ** *p*-value < 0.01; *** *p*-value < 0.005 for comparisons between DNFB-challenged mice fed control diet and comparison group using Student’s *t* test, and for comparisons between DMSO control samples and comparison groups.

Upon antigen-specific interactions with T cells, IL-12 production by antigen presenting cells induces the Th1 polarization of naïve CD4^+^ T cells [48,49], and stimulates IFN-γ production in CD4^+^ and CD8^+^ T cells [43,44]. IFN-γ-producing cells, including antigen-specific Th1 cells, are responsible for driving the recurrent elicitation phase of CHS [50]. Given the impact of BRB-E and PCA on IL-12 production during CHS, we analyzed IFN-γ production by T cells in secondary lymphoid organs during experimental DNFB-induced CHS, using flow cytometry (Figure S3A). Our flow cytometric analysis shows that a substantial decrease in IFNγ^+^ producing CD4^+^ T cells and CD8^+^ T cells in the lymph nodes and spleens of DNFB-challenged mice fed a BRB supplemented diet compared to those fed a control diet. Comparatively, DNFB-challenged mice fed the PCA supplemented diet saw only significant reductions in lymph nodal CD4^+^ IFNγ^+^ and splenic CD8^+^ IFNγ^+^ populations, relative to populations isolated from DNFB challenged mice fed the control diet (Figure 4A,B; Figure S3B,C). These intermediate effects on IFNγ production observed in DNFB-challenged PCA-fed mice compared to BRB-fed mice are reflective of the intermediate IL-12 down-regulation observed in vitro by dendritic cells and macrophages treated with PCA. This further demonstrates that different anthocyanin-derived and non-anthocyanin-derived phytochemicals in BRB also contribute to the down-regulation of the Th1 response during CHS.

Given the roles of natural killer (NK) cells in mediating CHS pathology [51], we also examined the impact of dietary supplementation with BRB and PCA on IFN-γ production by NK cells during DNFB induced CHS (Figure S4A). IFN-γ production by CD49b^+^CD3^-^ NK cells of the spleens and draining lymph nodes was reduced in DNFB challenged mice fed BRB, but not PCA, supplemented diet compared to DNFB challenged mice with control diet (Figure 4C; Figure S3D). We did not observe any differences in IFNγ production by NKT cells in DNFB-challenged mice fed BRB or PCA supplemented diets compared to mice fed the control diet (Figure 4D; Figure S3E). Since the dendritic cell production of IL-12 and CD80 activity is important for enhanced NK cell IFN-γ production and cytotoxicity [52,53], it is not surprising to observe reduced IFN-γ production in mice fed BRB supplemented diets.

Next, we determined the ability of lymph node and splenic T cells isolated from the different mouse groups to produce IFN-γ upon CD3 and CD28 stimulation in vitro. Cytokine ELISA demonstrated the decreased production of IFN-γ by CD3 stimulated T-cells isolated from the spleens and lymph nodes of DNFB-challenged mice fed PCA and BRB supplemented diets compared to DNFB challenged mice fed the control diet (Figure 4E,F). Taken together, our data demonstrate that dietary supplementation with BRB leads to the decreased expansion of IFN-γ producing lymphocytes and IFN-γ production in DNFB-mediated CHS. This supports our observations of BRB mediated down-regulation of IL-12 secretion by dendritic cells and macrophages. The PCA supplemented diet had an intermediate effect on IFN-γ production by T lymphocytes, compared to the BRB supplemented diet, demonstrating that the additional phytochemicals in BRB contribute to the observed effects. Interestingly, it has been shown that IL-12 synergizes with CD80-mediated antigen sensitization to expand CD8^+^ T cell activity [54], which could explain the more potent down-regulation of IFNγ^+^CD8^+^ T cells in DNFB-challenged mice fed a BRB supplemented diet relative to those fed a PCA supplemented diet.

### 3.5. Mediators of Effector Responses during CHS, Are Inhibited by BRB and PCA

Monocytes and macrophages are essential components of the CHS response and are recruited to the challenge site during the elicitation phase, where they are activated by Th1-associated IFN-γ to elicit CHS associated inflammation [55,56]. The IFN-γ mediated activation of macrophages and CD11b^+^Ly6C^hi^Ly6G^-^ monocytes results in the production of nitric oxide, an effector molecule that plays a critical role in mediating the pathology of CHS [57]. Indeed, therapies which target inducible nitric oxide synthase (iNOS) expression by macrophages have been shown to abrogate the CHS response to DNFB [58,59]. We, therefore, analyzed the effect of BRB and PCA on monocyte and macrophage populations and their effector responses during experimental CHS.

**Figure 5:**
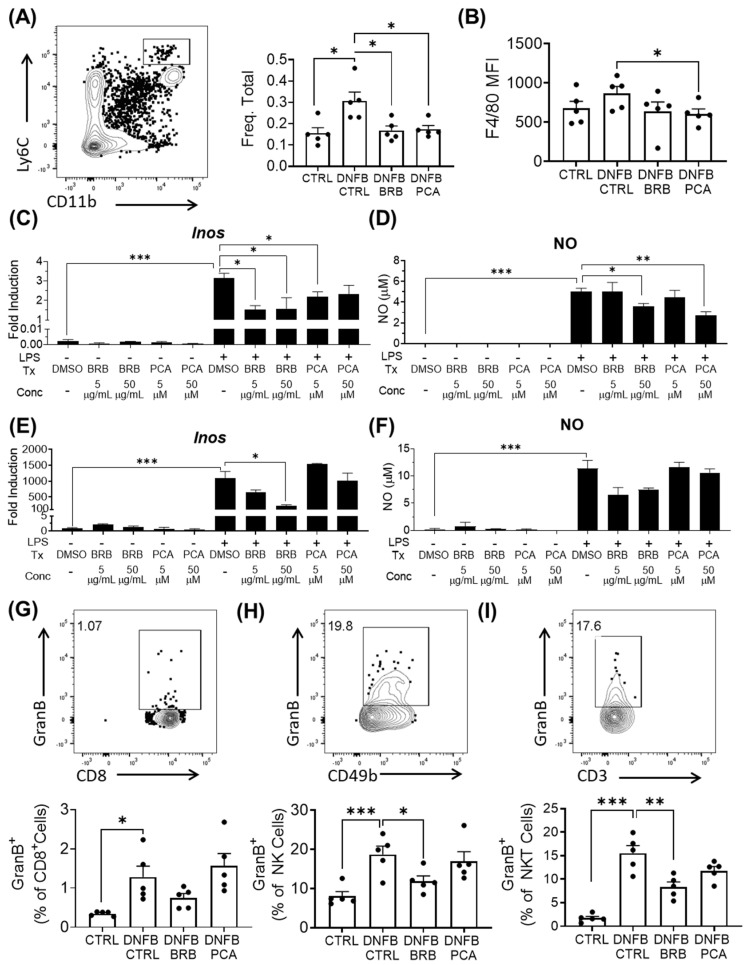
Mediators of effector responses during CHS are inhibited by BRB and PCA. (**A**) Frequency of CD11b^+^Ly6C^hi^Ly6G^-^ monocytes among total live cells in the spleen, determined by flow cytometry. A representative flow plot of the gated population is shown. (**B**) Mean fluorescent intensity (MFI) of F4/80 expression by CD11b^+^Ly6C^hi^ monocytes determined by flow cytometry. (**C**) Fold induction of *Nos2* expression in RAW264.7 cells at 24 h after incubation. (**D**) Nitric oxide production by RAW264.7 macrophages, 24 h after LPS stimulation. (**E**) Fold induction of *Nos2* expression by activation study bone marrow-derived dendritic cells at 24 h after LPS stimulation. (**F**) Nitric oxide production by activation study bone marrow-derived dendritic cells 24 h after LPS stimulation. (**G**–**I**) Frequencies of granzyme B production by lymph nodal (**G**) CD8^+^ T cells, (**H**) CD49b^+^CD3^−^ NK cells, and (**I**) CD49b^+^CD3^+^ NKT cells determined by flow cytometry. Representative flow plots of the gated populations are shown. * *p*-value < 0.05; ** *p*-value < 0.01; *** *p*-value < 0.005 for comparisons between DNFB-challenged mice fed control diets and comparison groups using Student’s *t* test, and for comparisons between DMSO control samples and comparison groups.

Monocytic CD11b^+^Ly6C^hi^Ly6G^−^ populations were reduced in the spleens of DNFB-challenged mice fed BRB and PCA supplemented diets, compared to challenged mice fed control diets (Figure 5A). In mice fed PCA supplemented diets, we also observed that this population showed a diminished expression of F4/80, a marker which has been implicated in sensitization to haptens [60,61] (Figure 5B). This down-regulation of F4/80 is a potential downstream effect of reduced IFNγ signaling in DNFB-induced CHS. To determine BRB and PCA mediated effects on the macrophage production of iNOS, we analyzed *iNOS* expression and nitric oxide production by LPS-stimulated RAW264.7 macrophages exposed to BRB-E, PCA or control media. BRB-E and PCA decreased *Nos2* expression and nitric oxide production in LPS stimulated macrophages (Figure 5C,D). Interestingly, BRB, but not PCA, decreased *Nos2* production and nitric oxide production by LPS-stimulated dendritic cells (Figure 5E,F). These results mimic BRB and PCA mediated effects on IL-12 production by activated macrophages and dendritic cells, suggesting that anthocyanin derived metabolites are likely primarily responsible for BRB mediated anti-inflammatory activity on macrophages. Previous BRB diet studies demonstrate that their anti-inflammatory effects may be attributed to down-regulation of iNOS [62,63]. BRB and PCA have also been shown to reduce the translation of iNOS in esophageal cells exposed to the carcinogen NMBA [28]. It is likely that the suppression of iNOS production contributes to the inhibitory effects of both BRB and PCA supplemented diets on CHS in vivo.

Granzyme B is an important effector in the elicitation phase of the CHS response and is present in the cytotoxic granules of NK cells and CTLs [64]. We therefore analyzed granzyme B production by CD8^+^ T cells and NK cells in draining lymph nodes of control mice and DNFB-challenged mice fed control, BRB or PCA supplemented diets. First, we did not observe any significant differences in granzyme B production in CD8^+^ T cells between DNFB-challenged mice fed control and BRB supplemented diets (Figure 5G; Figure S4A). However, among the lymph node NK and NKT cells of DNFB-challenged mice, we observed reduced granzyme B production in mice fed BRB, but not a PCA supplemented diet. (Figure 5H,I; Figure S4B,C). Taken together, our results suggest that the inhibition of granzyme B production by NK cells in BRB fed mice is likely not mediated by anthocyanin derived metabolites. As with IFN-γ production, there is evidence that the impact of BRB metabolites on dendritic cell IL-12 production plays a role [52]. The importance of NK cell effector activity as a major player in CHS pathology independent of T cells is demonstrated by studies showing that *Rag*^-/-^ mice are able to induce a potent CHS response through granzyme B and IFN-γ associated mechanisms [51]. Future studies characterizing BRB derived metabolites that regulate NK and NKT cell responses during CHS will provide additional candidates of CHS inhibition by BRB.

### 3.6. Il-17 Production Is Significantly Reduced by PCA Supplemented Diet, but Not BRB Supplemented Diet

**Figure 6:**
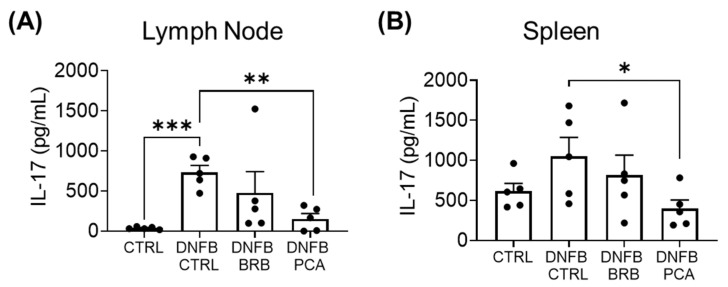
Il-17 production is significantly reduced by PCA supplemented diet, but not BRB supplemented diet. (**A**,**B**) IL-17 production (pg/mL) by CD3 stimulated cells isolated from the (**A**) draining lymph nodes and (**B**) spleens of experimental mice. * *p*-value < 0.05; ** *p*-value < 0.01; *** *p*-value < 0.005 for comparisons between DNFB-challenged mice fed control diets and comparison groups using Student’s *t* test.

IL-17 has been shown to play a critical role in CHS inflammation [65], where it works in tandem with IFN-γ to elicit the strongest CHS response [50]. In our DNFB model of CHS, we similarly observed an increase in IL-17 production in DNFB challenged mice (Figure 6A,B). We therefore analyzed the impact of diets supplemented with BRB and PCA on production of this cytokine by restimulated splenic and draining lymph node cells during DNFB-induced CHS. We observed a marked decrease in IL-17 production by T cells isolated form the spleens and lymph nodes of mice fed the PCA supplemented diet compared to mice fed the control diet, although this effect was not significant in mice fed a diet supplemented with BRB (Figure 6A,B). It has been previously reported that anthocyanins have a negative regulatory effect on IL-17 production by T cells in autoimmune disease [66]. Taken together with our observations on IFN-γ, our data demonstrate that PCA reduces IFNγ and IL-17 production by T cells, which have been shown to correlate with the reduced pathologies observed in previous CHS studies [45,50].

In summary, we demonstrate that the dietary intake of BRB and its anthocyanin metabolite PCA have an inhibitory effect on CHS. We also distinguish between PCA specific immunomodulatory effects and the global effects of the complex mixture of BRB phytochemicals on the pathways associated with CHS. Specifically, BRB impairs CHS initiation through the reduction of antigen sensitization by dendritic cells, through the reduction of CD80 and IL-12 production, while PCA preferentially inhibits monocyte and macrophage specific effector responses during CHS. Both BRB and PCA reduce IFN-γ associated type I immune responses and inhibit nitric oxide production by activated macrophages. However, BRB exerts a stronger inhibition on IFN-γ producing lymphocytes compared to PCA, which is likely, due to its effects on multiple antigen presenting cells. Our studies highlight the need for further study characterizing the diverse immunomodulatory phytochemicals that mediate BRB activity during CHS. Research remains ongoing on elucidating these mechanisms and determining the bioactivity of the metabolites identified to be inhibitors of CHS, in addition to anthocyanins and their metabolites, including PCA, as well as non-anthocyanin compounds and metabolites. Our findings are a major step towards unraveling the immunological mechanisms behind the BRB-mediated mitigation of CHS.

## Figures and Tables

**Table 1:** Composition of control, protocatechuic acid (PCA) and black raspberry (BRB) powder, containing pelleted murine diets.

Ingredient	Grams Per kg of Diet		
	AIN-76A (Control)	AIN-76A + PCA	AIN-76A + BRB
Casein	200	200	200
DL-methionine	3	3	3
Sucrose	500	449.5	450
Cornstarch	150	150	150
Corn oil	50	50	50
Cellulose	50	50	50
Mineral mix ^a^	35	35	35
Vitamin mix ^b^	10	10	10
Choline bitartrate	2	2	2
Protocatechuic acid ^c^	0	0.5	0.004
Freeze-dried black raspberry powder ^d^	0	0	50

^a^ as per AIN-76 mineral specifications: 5200 mg Cl, 4000 mg S, 3600 mg Mg, 1020 mg Fe, 1560 mg Cu, 337 mg Mn, 507 mg Zn, 35 mg Cr, 6 mg I, 54 mg Se, 30 mg Al, 2 mg F, 0,.2 mg Co, 0.1 mg B. ^b^ as per AIN-76 vitamin specifications: 6 mg thiamin HCl, 6 mg riboflavin, 7 mg pyridoxine HCl, 30 mg niacin, 16 mg calcium pantothenate, 2 mg folic acid, 0.2 mg biotin, 10 μg cyanocobalamin, 0.8 mg menadione sodium bisulfite, 4000 IU vitamin A palmitate, 50 IU vitamin E acetate, 1000 IU vitamin D3. ^c^ estimated based on previous studies [32]. ^d^ contained 1387 mg total anthocyanins/kg diet or 3.08 mmol total anthocyanins/kg diet.

## Supplementary Materials

The following are available online at https://www.mdpi.com/2072-6643/12/6/1701/s1, Figure S1. (A) Percent change in mouse weight from initial weight of mice fed experimental diets over the course of 12 weeks. (B) Average food consumption measured weekly by groups of mice fed CTRL, BRB supplemented, or PCA supplemented diet (n = 20 per group) over the course of 12 weeks. (C) The timeline of animal handling for the CHS experiment. Mice were fed experimental diets for a period of 3 weeks before abdominal sensitizations with DNFB at days 0, 4, and 7. Mice were challenged aurally on the left ear with DNFB to induce CHS at days 14 and 17. At day 18, mice were sacrificed and samples were collected for downstream analysis. Figure S2. (A) The gating strategy used to determine CD11c+ dendritic cell populations by flow cytometry in both the draining lymph nodes and spleens of experimental mice. (B) Representative comparative histograms of CD80, CD86, and MHCII expression by splenic CD11c+ dendritic cells of experimental mice.
