# Supplementary material for: Black Raspberries and Protocatechuic Acid Mitigate DNFB-Induced Contact Hypersensitivity by Down-Regulating Dendritic Cell Activation and Inhibiting Mediators of Effector Responses
Source: Nutrients. 2020 Jun 6;12(6):1701. doi: 10.3390/nu12061701 (PMC7352349; doi:10.3390/nu12061701)
Supplement: Supplementary file 1 [file nutrients-12-01701-s001.pdf]

Supplementary Material

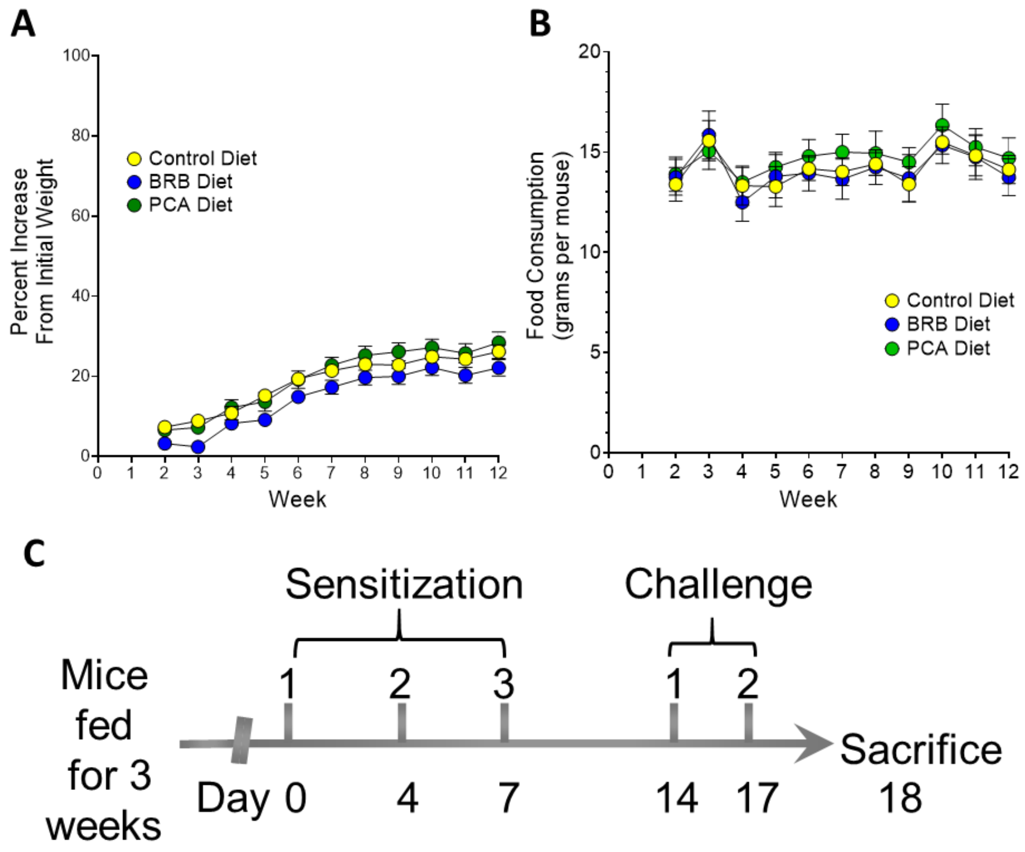

Figure S1. (A) Percent change in mouse weight from initial weight of mice fed experimental diets over the course of 12 weeks. **(B)** Average food consumption measured weekly by groups of mice fed CTRL, BRB supplemented, or PCA supplemented diet (n= 20 per group) over the course of 12 weeks. **(C)** The timeline of animal handling for the CHS experiment. Mice were fed experimental diets for a period of 3 weeks before abdominal sensitizations with DNFB at days 0, 4, and 7. Mice were challenged aurally on the left ear with DNFB to induce CHS at days 14 and 17. At day 18, mice were sacrificed and samples were collected for downstream analysis.

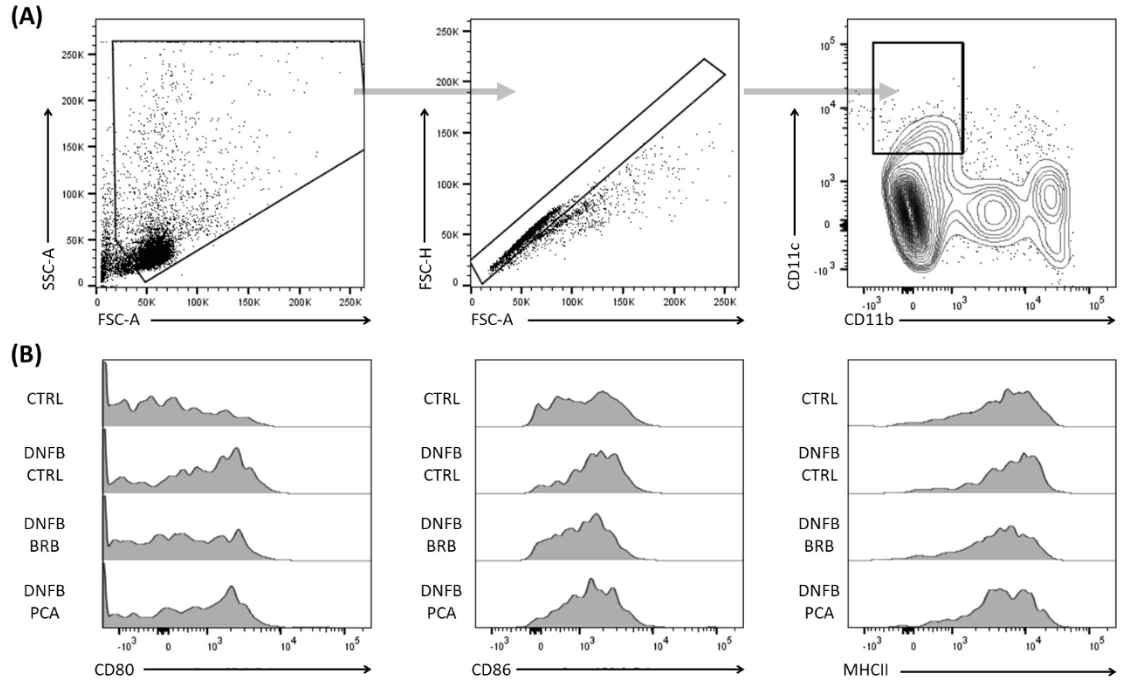

Figure S2. (A) The gating strategy used to determine CD11c⁺ dendritic cell populations by flow cytometry in both the draining lymph nodes and spleens of experimental mice. **(B)** Representative comparative histograms of CD80, CD86, and MHCII expression by splenic CD11c⁺ dendritic cells of experimental mice.

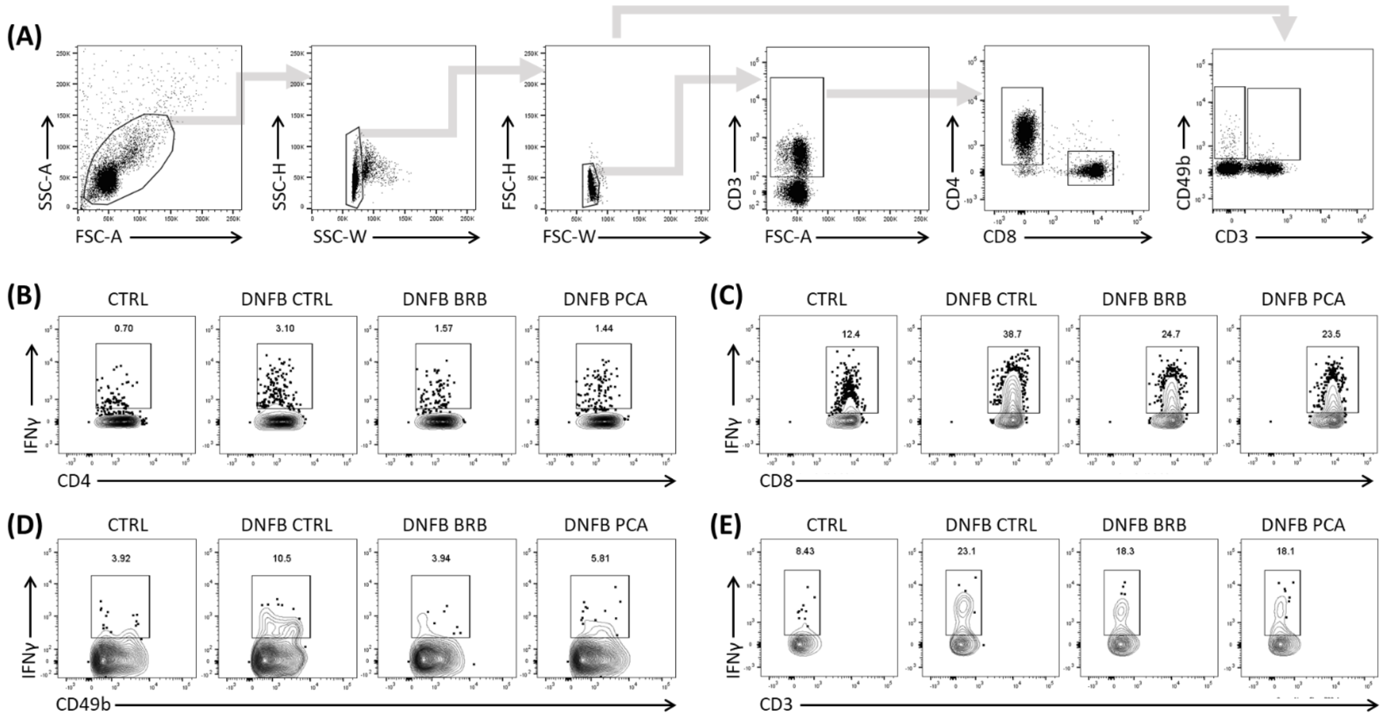

Figure S3. (A) The gating strategy used to determine $CD4^+$ T-cell, $CD8^+$ T-cell, $CD49b^+CD3^-$NK cell, and $CD49b^+CD3^+$ NKT cell populations by flow cytometry in both the draining lymph nodes and spleens of experimental mice. **(B-E)** Representative flow cytometry plots of IFNγ expression by (B) $CD4^+$ T-cell, (C) $CD8^+$ T-cell, (D) $CD49b^+CD3^-$ NK cell and (E) $CD49b^+CD3^+$ NKT cell populations in the draining lymph nodes of experimental mice.

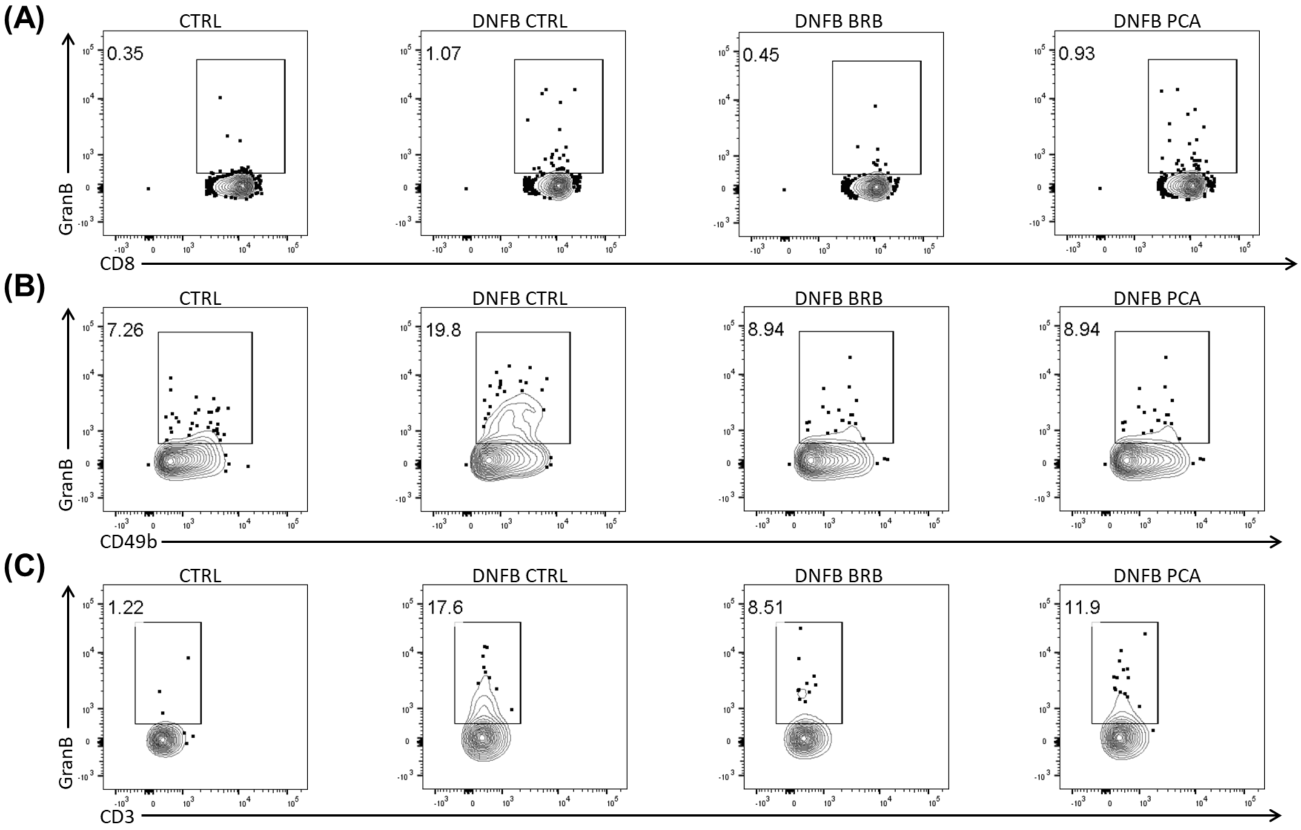

Figure S4. (A-C) Representative flow cytometry plots of granzyme B expression by (A) $CD8^+$ T-cell, (B) $CD49b^+CD3^-$ NK cell and (C) $CD49b^+CD3^+$ NKT cell populations in the draining lymph nodes of experimental mice.
